# PTSD Coach Version 3.1: A Closer Look at the Reach, Use, and Potential Impact of This Updated Mobile Health App in the General Public

**DOI:** 10.2196/34744

**Published:** 2022-03-29

**Authors:** Haijing Wu Hallenbeck, Beth K Jaworski, Joseph Wielgosz, Eric Kuhn, Kelly M Ramsey, Katherine Taylor, Katherine Juhasz, Pearl McGee-Vincent, Margaret-Anne Mackintosh, Jason E Owen

**Affiliations:** 1 Dissemination and Training Division National Center for Posttraumatic Stress Disorder Veterans Affairs Palo Alto Health Care System Menlo Park, CA United States; 2 Department of Psychiatry and Behavioral Sciences Stanford University School of Medicine Stanford, CA United States; 3 Sierra Pacific Mental Illness Research Education and Clinical Center Veterans Affairs Palo Alto Healthcare System Palo Alto, CA United States

**Keywords:** posttraumatic stress disorder, trauma, mental health, mHealth, mobile app, public health, self-management, mobile phone

## Abstract

**Background:**

With widespread smartphone ownership, mobile health apps (mHealth) can expand access to evidence-based interventions for mental health conditions, including posttraumatic stress disorder (PTSD). Research to evaluate new features and capabilities in these apps is critical but lags behind app development. The initial release of PTSD Coach, a free self-management app developed by the US Departments of Veterans Affairs and Defense, was found to have a positive public health impact. However, major stakeholder-driven updates to the app have yet to be evaluated.

**Objective:**

We aimed to characterize the reach, use, and potential impact of PTSD Coach Version 3.1 in the general public. As part of characterizing use, we investigated the use of specific app features, which extended previous work on PTSD Coach.

**Methods:**

We examined the naturalistic use of PTSD Coach during a 1-year observation period between April 20, 2020, and April 19, 2021, using anonymous in-app event data to generate summary metrics for users.

**Results:**

During the observation period, PTSD Coach was broadly disseminated to the public, reaching approximately 150,000 total users and 20,000 users per month. On average, users used the app 3 times across 3 separate days for 18 minutes in total, with steep drop-offs in use over time; a subset of users, however, demonstrated high or sustained engagement. More than half of users (79,099/128,691, 61.46%) accessed one or more main content areas of the app (ie, Manage Symptoms, Track Progress, Learn, or Get Support). Among content areas, features under Manage Symptoms (including coping tools) were accessed most frequently, by over 40% of users (53,314/128,691, 41.43% to 56,971/128,691, 44.27%, depending on the feature). Users who provided initial distress ratings (56,971/128,691, 44.27%) reported relatively high momentary distress (mean 6.03, SD 2.52, on a scale of 0-10), and the use of a coping tool modestly improved momentary distress (mean −1.38, SD 1.70). Among users who completed at least one PTSD Checklist for DSM-5 (PCL-5) assessment (17,589/128,691, 13.67%), PTSD symptoms were largely above the clinical threshold (mean 49.80, SD 16.36). Among users who completed at least two PCL-5 assessments (4989/128,691, 3.88%), PTSD symptoms decreased from the first to last assessment (mean −4.35, SD 15.29), with approximately one-third (1585/4989, 31.77%) of these users experiencing clinically significant improvements.

**Conclusions:**

PTSD Coach continues to fulfill its mission as a public health resource. Version 3.1 compares favorably with version 1 on most metrics related to reach, use, and potential impact. Although benefits appear modest on an individual basis, the app provides these benefits to a large population. For mHealth apps to reach their full potential in supporting trauma recovery, future research should aim to understand the utility of individual app features and identify strategies to maximize overall effectiveness and engagement.

## Introduction

### Background on PTSD Coach

With 85% of US adults now owning a smartphone [1], mobile health (mHealth) apps remain one of the most promising avenues for disseminating evidence-based interventions for mental health [2]. Increased dissemination of mental health interventions is sorely needed, as only a minority of individuals with mental health concerns receive services. For example, only one-third of US adults with a moderately severe mental health condition received treatment in a given year [3]. There are a variety of barriers to traditional, in-person services, including mental health stigma and limited access to care, both of which are more pronounced for racial and ethnic minorities [4,5]. mHealth apps are well-positioned to mitigate these barriers, with their discreet nature and a similar rate of smartphone ownership across White, Black, and Hispanic groups in the United States [1]. Responding to this apparent potential, the development of mHealth apps has exploded in popularity; however, systematic research on them has lagged far behind [6-8]. To understand their value from a public health perspective, it is important to characterize the reach, use, and potential benefits of these apps. Furthermore, evaluating mHealth apps on an ongoing basis, especially as apps are modified or updated with new features, can provide an important feedback loop to inform researchers about what is working well and what could be improved within apps [9]. This study focuses on assessing the public health impact of an mHealth app for posttraumatic stress disorder (PTSD), with substantial updates to both the features in the app and the analytic capabilities for understanding its use.

PTSD is a significant mental health condition due to its often debilitating effects on psychosocial functioning and quality of life [10,11] and its high prevalence, especially among military veterans [12,13]. As part of a portfolio of mHealth apps [14], the PTSD Coach app was developed by the US Department of Veterans Affairs (VA) National Center for PTSD (NCPTSD) and the US Department of Defense Center for Telehealth and Technology. PTSD Coach was designed with veterans and service members in mind and was also intended as a public health resource to help any individual impacted by trauma. As such, the app has been available to the public since 2011 on both iOS and Android platforms. Drawing from evidence-based treatments (eg, cognitive behavioral therapy [15]), PTSD Coach provides psychoeducation, self-assessments, coping tools, and resources for support and professional care; the self-management app is not meant to replace treatment with a mental health professional. Importantly, PTSD Coach is provided free of charge and protects users’ privacy by collecting data anonymously (ie, no identifying information). The app is offered in both English and Spanish, is accessible to people with visual and hearing impairments, and can be used without internet connectivity. PTSD Coach is available worldwide, through its US version (described in this paper) as well as separate versions developed in 6 other countries (a result of sharing source code with international partners [16]).

In 2015, Owen et al [17] sought to provide an initial characterization of the reach, use, and potential impact of PTSD Coach in the general public. To do so, they examined *in the wild* data (ie, data from people who are using the publicly available version of the app in their everyday lives), thus enabling the assessment of naturalistic patterns of use. Evaluating PTSD Coach, version 1, between March 2011 and February 2014, the authors found that the app had been broadly disseminated with over 150,000 downloads and over 10,000 active users per month, had reached its target audience (eg, veterans and civilians with PTSD symptoms, their family members, and mental health providers), and was reviewed positively by users. The authors provided descriptive statistics on patterns of app use; for example, showing that users used the app an average of 6 times and for a total duration of 5 minutes. Of note, although most users had steep drop-offs in use of the app over time, as is the case with self-management apps more broadly [18], there was a subset of high-engagement users who reported that they incorporated the app as part of a daily routine and continued to use the app even a year later. Lastly, on average, users who completed self-assessments endorsed PTSD symptoms above clinical threshold and rated high levels of momentary distress; after using a coping tool, momentary distress decreased by an average of 2 points (on a scale of 0-10), highlighting the benefits of the app during times of need. Findings from the study by Owen et al [17] are consistent with findings from controlled research studies (eg, randomized controlled trials) on PTSD Coach, in which the app was associated with positive user experiences [19,20] and benefits [21-23] in both veteran and civilian samples.

### Updates to PTSD Coach

**Figure 1:**
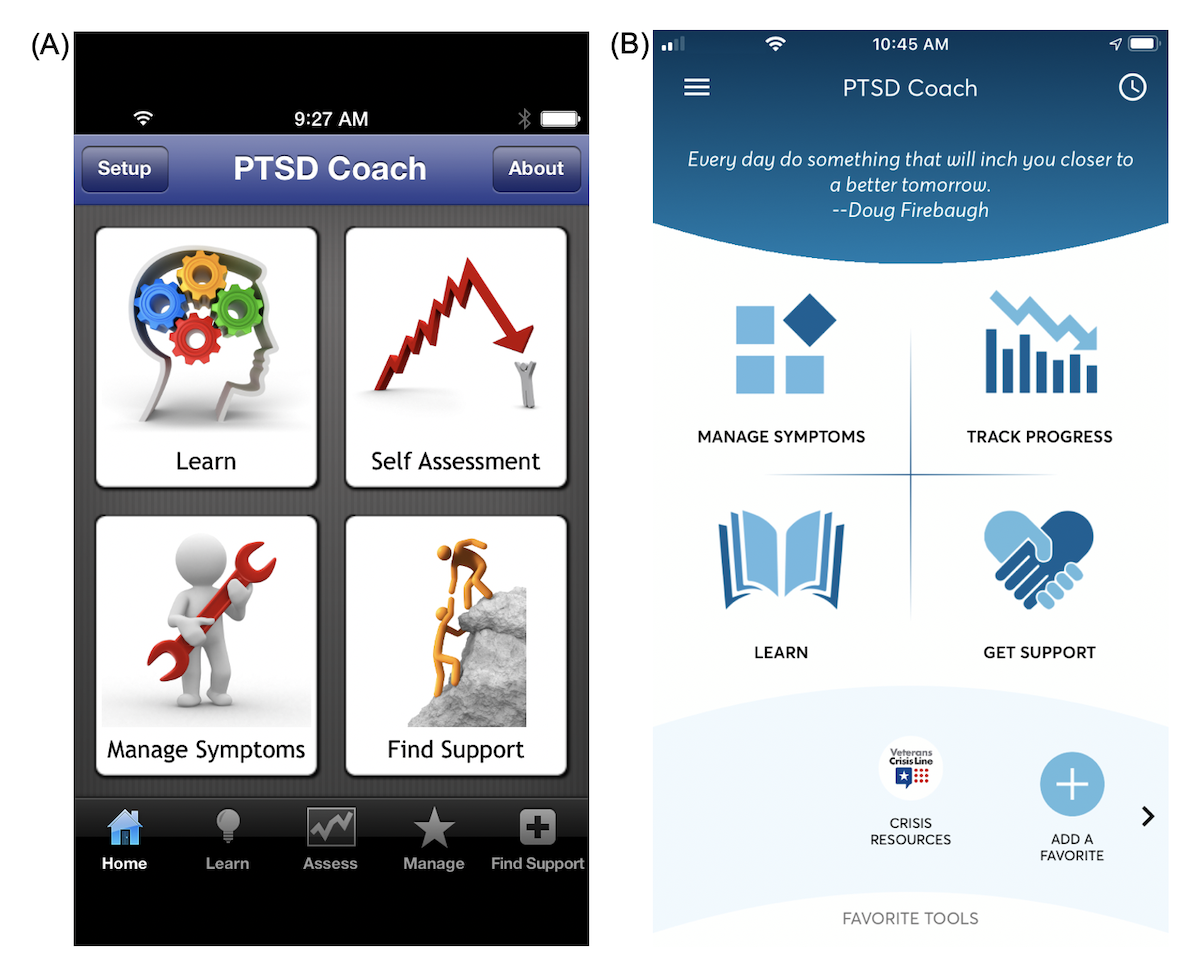
Screenshots of the PTSD Coach home screen with 4 main content areas: (A) home screen from version 1; (B) home screen from version 3.1 (current version).

Since the study by Owen et al [17] was published, the PTSD Coach app has undergone several substantial updates to its design and features to address stakeholder feedback from users and health care professionals. The look and feel of PTSD Coach was revamped to have a clean, modern design (see Figures 1A and 1B as well as 2A and 2B for examples), and the app incorporated updated information about PTSD that was consistent with the Diagnostic and Statistical Manual of Mental Disorders, 5th Edition [24]. In addition, new evidence-based therapeutic features were added to the app, including mindfulness and relationship tools and the option to complete a safety plan for suicide prevention. For increased convenience, users can now access favorite tools and view an inspiring quote on the home screen of the app. Users can also more easily share the app with family and friends and provide feedback about the app to the development team. Finally, the app’s development team has improved its ability to detect, track, and resolve problems in the app on both the iOS and Android platforms. This may be especially critical for Android, which has substantial heterogeneity in its smartphones and had stability issues with its earlier operating system versions. In the previous evaluation of version 1, Android users reviewed the app less positively and were found to have lower rates of use and smaller benefits compared with iOS users [17].

**Figure 2:**
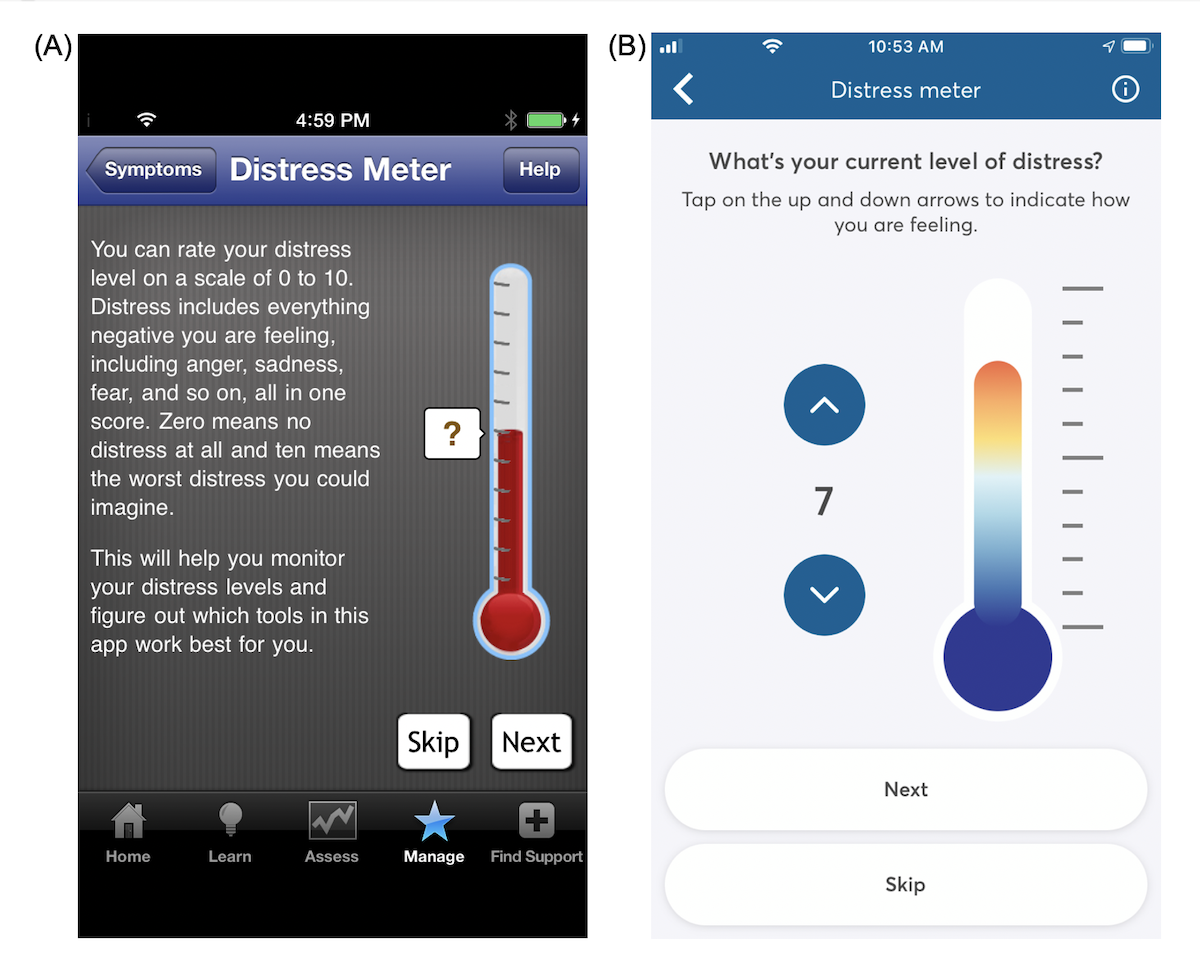
Screenshots of the PTSD Coach distress meter with which users can rate subjective units of distress on a scale of 0 to 10 rating: (A) distress meter from version 1; (B) distress meter from version 3.1 (current version).

Another significant update is that PTSD Coach can now accommodate user-level analyses on all features of the app. To do so, anonymous install codes (generated upon the first launch of the app) are used as a proxy for individual users. Previously, to establish a baseline on the reach, use, and potential impact of PTSD Coach in the general public, Owen et al [17] analyzed data which were available in aggregate form only (ie, at the group level). The data were primarily available in aggregate form for two groups: first-time users and returning users. It was not possible to link which first-time users went on to become returning users; consequently, they could not analyze any individual user’s data (eg, change in PTSD symptoms over time). Furthermore, some metrics (eg, levels of momentary distress) were not available to be analyzed for users as a whole (ie, first-time and returning users combined), and other metrics (eg, use of specific coping tools) were not available at all. Thus, this limited obtaining detailed information about how users interacted with the app. Having this information could inform researchers of what users’ needs are (eg, most frequently reported PTSD symptoms) or what users finding helpful in the app (eg, most frequently used coping tools).

### Objectives of This Study

In this study, we sought to characterize the reach, use, and potential impact of the current version of PTSD Coach, version 3.1, which was released on April 20, 2020. In particular, we aimed to build upon the study by Owen et al [17] by characterizing the use of specific features of the app, within the main content areas, to obtain a fine-grained picture of how users are interacting with the app. This aim is consistent with the growing recognition in the literature that evaluating the *quality* of engagement with an app is critical in addition to the *quantity* of engagement [25]. Given the differences observed between iOS and Android users with version 1 of the app, we also compared reach, use, and impact by platform. Notably, we did not seek to directly replicate Owen et al [17] because of changes in the instrumentation of the app (eg, differences in the availability of data), mismatch in the length of data observation periods (ie, 3 years vs 1 year), and relevant environmental factors that we could not control (eg, increasing proliferation of smartphone devices). However, given the updates to the PTSD Coach app, we expected that the findings for version 3.1 would generally demonstrate maintained or improved metrics related to reach, use, and potential impact, compared with version 1.

Regarding relevant environmental factors, we note that our data observation period (April 2020 to April 2021) fell squarely within the timeframe of the COVID-19 pandemic. Because the pandemic contributed to rising levels of mental health symptoms [26], it was possible that we would observe evidence of wider reach and increased use of PTSD Coach as a function of the stressors associated with the pandemic. Because we did not have version 3.1 data before April 2020, we could not rule out this possibility, and we certainly hope that the app has been helpful to those experiencing heightened PTSD symptoms during this challenging time. In this evaluation, we consider the potential role of the pandemic by contextualizing our findings with this lens (eg, seeing whether users are endorsing higher levels of PTSD symptoms now compared with in the study by Owen et al [17] and seeing whether use of the app seemed to fluctuate alongside peaks of COVID-19 cases in the country).

Our data observation period also overlapped with 2 large-scale initiatives within the VA health care system. First, the VA Office of Connected Care (OCC) started a program in 2016 that distributes tablets to veterans engaging in telehealth services; this program is ongoing and, more recently, has focused on iOS tablets specifically [27,28]. Between April 2020 and April 2021, the OCC downloaded PTSD Coach along with other health care–related apps onto 95,000 iOS tablets provided to veterans. Second, between January 2020 and December 2020, the NCPTSD trained 1100 VA staff members at 19 sites across the country on the use of VA mHealth apps in the care of veterans [29]. As part of these training sessions, VA staff downloaded and explored the PTSD Coach app, including a safety plan for suicide prevention. Because of the anonymous nature of the app data, we were unable to determine which PTSD Coach users were veterans using VA-provided iOS tablets or VA staff members undergoing mHealth app training. However, we discuss aspects of our findings that were likely shaped by these initiatives.

## Methods

### Data Sources

We utilized download and use data from the public version of the PTSD Coach mobile app between April 20, 2020, and April 19, 2021. The download data came from the Apple App Store (for iOS devices) or the Google Play Store (for Android devices) and included the country code associated with a user’s Apple ID or Google Play account. The use data corresponded to in-app events, that is, actions taken by the user in the app (eg, screens selected and buttons pressed), and were collected for quality improvement purposes. There were use data for each install code, which was a unique, random sequence of numbers and letters generated upon the first launch of the app. These install codes were used as proxies for individual app users. Although we do not think this was the case for most users, a user could have had more than one install code as a result of deleting and installing the app multiple times or installing the app on multiple devices. Users can also opt out of sharing their use data within the app settings. All use data were anonymous and encrypted and stored on a secure server. No identifying or device information (other than whether the platform was iOS or Android) was collected or stored.

### Ethics Approval

These data were collected by the NCPTSD mobile mental health program as part of ongoing quality improvement, which was approved by the Palo Alto VA Research and Development Committee (RDIS No. ROS0021). The Institutional Review Board at Stanford University School of Medicine reviewed the project and determined that it was non-research.

### The PTSD Coach, Version 3.1, Mobile App

#### Onboarding and Home Screen

After users accepted the End User License Agreement (EULA), they were shown a brief tutorial about the four main content areas of the app: Manage Symptoms, Track Progress, Learn, and Get Support. Then, they could opt to personalize the app (eg, by adding pictures, music, support contacts, or switching to using the app in Spanish) or they could proceed directly to the app content (which took them to the home screen). From the home screen, users could access the 4 main content areas (Figure 1B). In addition, from the home screen, users could open a lateral menu with the option of completing a safety plan for suicide prevention. This lateral menu also had options for users to learn more about and personalize the app, manage their data, and share and give feedback about the app.

#### Manage Symptoms

Users could indicate a current PTSD-related symptom that they were experiencing: Reminded of Trauma, Avoiding Triggers, Disconnected From People, Disconnected From Reality, Sad/Hopeless, Worried/Anxious, Angry, and Unable to Sleep. Users could access a coping tool either through a recommendation based on a selected symptom or by viewing the complete list of tools; they could also access a list of tools previously marked as favorites. There were a total of 23 coping tools (see Multimedia Appendix 1 for the list). Before and after using a tool, users were asked to rate their current level of distress (ie, momentary distress) using a visual thermometer analog corresponding to a scale from 0 to 10 (Figure 2B); users had the option to skip or turn off this rating feature. We refer to these ratings as pretool and posttool subjective units of distress (SUDs).

#### Track Progress

Users could complete and receive feedback on a self-assessment of their PTSD symptoms, view a graph and details of their past self-assessments, and set a reminder to take future self-assessments. The self-assessment used was the PTSD Checklist for DSM-5 (PCL-5 [30]), which has 20 items that are answered on a 5-point scale (0=*not at all* to 4=*extremely*) about how much a person was bothered by individual symptoms in the past month. The PCL-5 was found to have good reliability and validity in both civilian [31] and veteran [32] samples. Scores of 31 to 33 or higher correspond with a likely PTSD diagnosis [32], and decreases of approximately ≥5 points and ≥10 points indicate reliable and clinically significant change, respectively [30].

#### Learn

Users could read psychoeducational information organized under 3 categories: About PTSD, Getting Professional Help, and PTSD and the Family. There are 21 learn topics under About PTSD, 22 learn topics under Getting Professional Help, and 12 learn topics under PTSD and the Family (see Multimedia Appendix 1 for the list).

#### Get Support

Users could access resources for additional support organized under 3 categories: Crisis Resources, Find Professional Care, and Grow Your Support. Under Crisis Resources, information for suicide prevention and crisis hotlines were included, as well as the option to add a personal support contact. Under Find Professional Care, a broad range of mental health treatment resources were listed, including information for military-specific treatment options (eg, VAs and Vet Centers) and options open to the general public (eg, Psychology Today). Under Grow Your Support, there were ideas about ways to reach out to and connect with others, including information for joining both military-specific (eg, Team Red, White, and Blue) and general groups (eg, Meetup groups). Resources under all 3 categories contained direct links to phone numbers and websites.

#### Safety Plan for Suicide Prevention

Located in the lateral menu, the safety plan for suicide prevention was based on the Safety Planning Intervention [33]. In a tutorial, users were first oriented to the purpose of the safety plan, encouraged to discuss the safety plan with a provider, and given crisis resources and options for finding professional care. The safety plan was divided into 6 sequential steps, which comprised a predetermined and individualized set of strategies designed to help individuals manage mental health crises (eg, suicidal urges) instead of acting on impulse. These steps correspond to identifying (1) warning signs, (2) self-coping strategies, (3) social contacts and settings for purposes of distraction, (4) family and friends for purposes of crisis management, (5) mental health professionals and agencies, and (6) ways to restrict access to lethal means. Steps 3 and 6 have 2 parts, and steps 3 through 6 involve adding a contact. For analysis, a safety plan was considered complete if a user filled out complete information for all 6 steps.

### Analysis Plan

There were 9,415,339 in-app events in the data observation period between April 20, 2020, and April 19, 2021. Each *event* contained an event name, date and time stamp, and the associated user’s install code and platform (ie, iOS or Android), as well as any nontext data entered by the user (eg, SUDs ratings). Following Kozlov et al [34], we considered events with time stamps within 30 minutes of one another as part of a single *visit* to the app; we took this approach to avoid inconsistencies in the default app instrumentation for marking the end of a visit. Thus, visit duration reflected the time that passed between the first and last events that belonged to the same visit. If a user had only one event in total (the result of opening the app without responding to the EULA), visit duration was encoded as 0 minutes; this was also the case for total app use duration and time between the first and last app use.

Data preprocessing was conducted using Python (version 3.7.7; Python Software Foundation) with the pandas (version 1.05) [35] and pyodbc (version 4.0.0) [36] libraries. Event data were extracted from the server and labeled with their corresponding visit numbers via Python scripts with embedded SQL queries. Next, the data were run through a second Python script to generate per-user summaries. This script first cleaned the data and removed duplicate events, then extracted user-level metrics in a table format and combined the user-level metrics into a unified data set. We used the final data set of user-level metrics to run descriptive statistics and difference tests using SPSS Statistics (version 26; IBM) to evaluate the reach, use, and potential impact of PTSD Coach, for all users and then separately for iOS and Android users. For difference tests, the *t* test (2-tailed) effect size was Cohen *d* (small: 0.2, medium: 0.5, and large: 0.8), and the chi-square test effect size was Cramer *V* (small: 0.1 to <0.3, medium: 0.3 to <0.5, and large: ≥0.5). Variables that were not normally distributed were Box-Cox transformed (*λ*=−0.3, with an additional 0.001 constant added to handle values of 0) before performing difference tests and calculating effect sizes.

For *reach*, we examined numbers of downloads and users, including number of active users per month (ie, users who used the app at least once during a given month) to see if reach was sustained over the data observation period. For *use*, we examined overall use of the app (eg, total number of visits), use of the app over time (eg, retention), and use of specific features of the app (eg, whether a coping tool was accessed). Regarding use of specific app features, we determined the most frequently used features at the level of the user, in which the number of users who accessed a specific app feature at least once (collapsing across all visits) was divided by the total number of users. For *impact*, we examined first SUDs ratings and first PCL-5 scores as well as changes in these metrics, by subtracting posttool SUDs ratings from pretool SUDs ratings and by subtracting the last PCL-5 score from the first PCL-5 score. To accurately characterize *use* and *impact*, we limited analyses to install codes whose first event fell inside the observation period; we considered these to be new users who started using the app after version 3.1 was released and for whom we had maximum use data.

Lastly, regarding use of the app over time, for calculating retention, we divided the 12-month period into the following bins: days 1 to 7, weeks 1 to 4, and months 1 to 12. The starting point was the user’s first event. Subsequent events after the first event could fall into different bins—for example, ≥0 and <24 hours after the first event (day 1), ≥0 and <7 days after the first event (week 1), and ≥0 and <30 days after the first event (month 1). For the user percentage calculation for each bin, a user was included in the numerator if they had a qualifying subsequent event relative to their first event. The denominator was adjusted for each bin to reflect the number of users with potential observable data, which was based on when users started using the app. For example, a user whose first event occurred on the first day of the observation period (April 20, 2020) would be included in the denominator for all bins. In contrast, a user whose first event occurred on the second-to-last day of the observation period (April 18, 2021) would be included in the denominator for the day 1 bin only, as it was not possible to further observe their data with the observation period cutoff (April 19, 2021). We evaluated both classic retention (ie, app use on a specific day relative to first use) and rolling retention (ie, app use on or after a specific day, relative to first use).

## Results

### All Users

#### Reach

**Figure 3:**
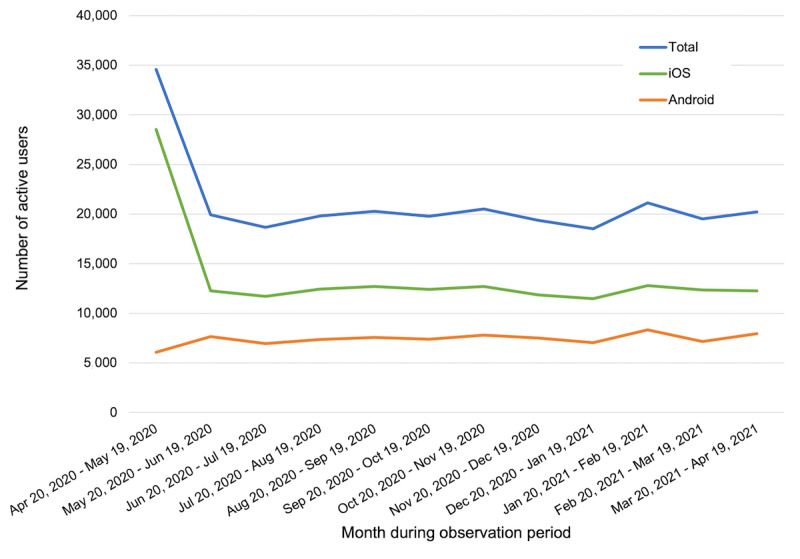
Reach of PTSD Coach, version 3.1, between April 20, 2020, and April 19, 2021.

During the observation period between April 20, 2020, and April 19, 2021, there were 207,001 downloads of the PTSD Coach app. Most of these (188,203/207,001, 90.92%) were downloads from user accounts based in the United States. There were 148,354 app users (ie, users who used PTSD Coach at any point during the observation period) and an average of 21,032 active monthly users (ie, users who used PTSD Coach at least once during a given month). The number of active monthly users per month during the observation period is shown in Figure 3. This number was highest in the first month, fell in the second month, and remained relatively steady for the remainder of the year—a pattern that was driven by the number of iOS users (see the section *Reach* under the subheading *Comparison of iOS and Android Users* below for an explanation). Among the 148,354 total users, 128,691 (86.74%) were new users (ie, they started using the app during the observation period).

#### Use

##### Overall Use

The metrics of overall use for new users of PTSD Coach are presented in Table 1. On average, users of PTSD Coach visited the app approximately 3 times, with each visit having an average duration of 5 minutes and involving 18 events. In total, users spent approximately 18 minutes using the app across 3 unique days. Compared with these means, the medians and modes were lower, indicating that the means were positively skewed by extreme values; indeed, maximum values were much larger than the means, whereas IQRs remained relatively small. These findings illustrate that there was a subset of users with much higher levels of engagement than the average user. For example, 2.02% (2601/128,691) of new users visited the app on average ≥18 times (ie, ≥2 SDs above the mean for all new users), corresponding to a total of approximately 230 minutes spent using the app across 26 unique days.

**Table 1:** Overall use of PTSD Coach, version 3.1, among new users (n=128,691) between April 20, 2020, and April 19, 2021.

Category	Mean (SD)	Median^a^	Mode^a^	Range	IQR^a^
Number of visits	3.26 (7.41)	2	1	1.00-501.00	2
Number of events per visit	17.89 (25.70)	9	1	1.00-1052.00	21
Visit duration (minutes)	4.60 (7.14)	2	0	0.00-203.20	6
Total duration (minutes)	17.55 (58.62)	4	0	0.00-6472.37	16
Number of unique days	2.70 (4.88)	1	1	1.00-222.00	2

^a^This was calculated after rounding values to the nearest whole integer for the number of events per visit, visit duration, and total duration.

##### Use Over Time

**Figure 4:**
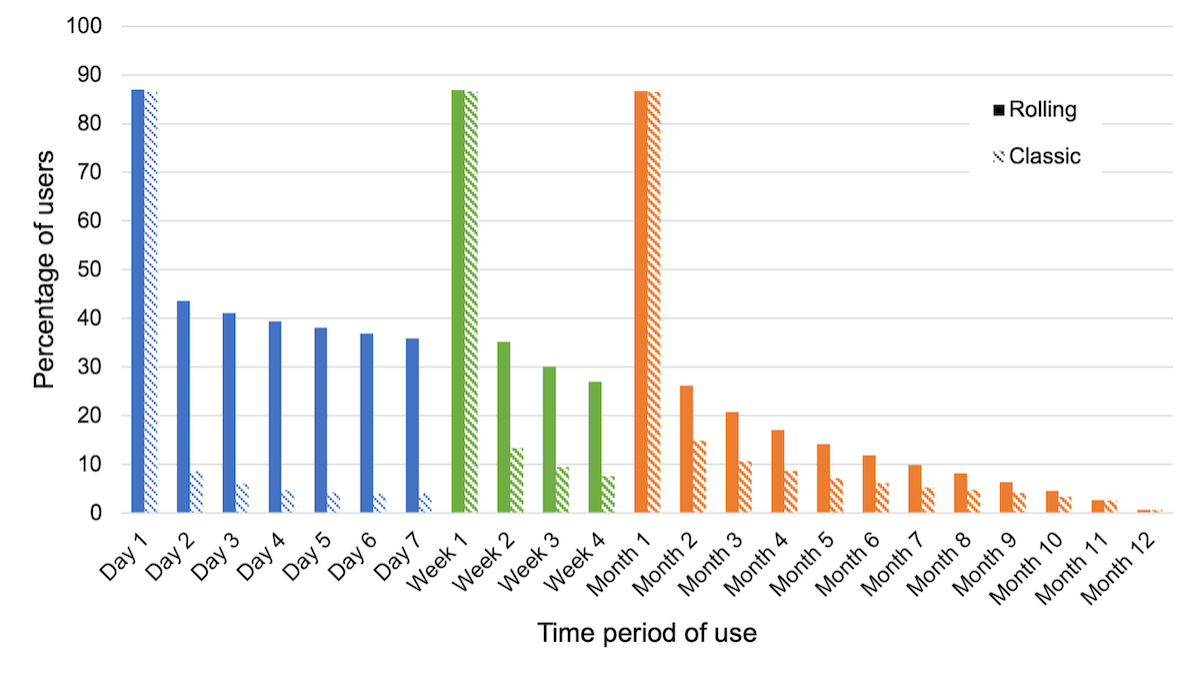
Rolling and classic retention of PTSD Coach, version 3.1, new users.

The rates of classic retention (app use on a specific day, relative to first use) and rolling retention (app use on or after a specific day, relative to first use) for new users during the 12-month observation period are displayed in Figure 4. As expected, rolling retention rates were higher than classic retention rates. For both classic and rolling retention, approximately 87% of users (110,971/128,305, 86.49% and 111,660/128,305, 87.03%, respectively) used the app during the first day of opening it (day 1). Because week 1 and month 1 are inclusive of day 1, retention rates across these 3 periods are very similar. Beyond day 1, 8.53% (10,923/128,032) of users used the app during day 2 (classic retention), and 43.54% (55,744/128,032) of users used the app during day 2 or later (rolling retention). At the other extreme, for month 12, classic and rolling retention were identical because of the observation period cutoff, with 0.69% (62/8953) of users using the app in the 12th month after the initial opening of the app. Lastly, relevant to rolling retention specifically, new users had on average 31.54 (SD 64.01) days that spanned their first and last use of the app. Upon restriction to the subset of high-engagement users identified above (2601/128,691, 2.02% of new users), the average time between the first and last use of the app was 173.76 (SD 96.91) days.

##### Use of Specific Features

After accepting the EULA and completing onboarding, 65.86% (84,763/128,691) of new users arrived at the home screen. A total of 61.46% (79,099/128,691) of users visited a content area. Specifically, 25.69% (33,057/128,691) of the users visited 1 content area, 13% (16,702/128,691) visited 2 content areas, 9.58% (12,325/128,691) visited 3 content areas, and 13.22% (17,015/128,691) visited all 4 content areas. The mean number of content areas visited was 1.33 (SD 1.41).

First, regarding specific content areas visited, 55.68% (71,649/128,691) of users visited the Manage Symptoms. Within this content area, 43.78% (56,347/128,691) of users selected at least one of the eight PTSD-related symptoms. The three most frequently selected symptoms were Reminded of Trauma (29,960/128,691, 23.23%), Avoiding Triggers (19,387/128,691, 15.06%), and Unable to Sleep (18,127/128,691, 14.08%). Also within this content area, 41.43% (53,314/128,691) of users accessed at least one coping tool. The three most frequently accessed tools were Change Your Perspective (19,988/128,691, 15.53%), Inspiring Quotes (19,668/128,691, 15.28%), and Ambient Sounds (17,753/128,691, 13.8%). Providing ratings of momentary distress, 44.27% (56,971/128,691) of users rated their SUDs before using a coping tool, and 13.86% (17,841/128,691) of users rated their SUDs before and after using a tool.

Second, 27.57% (35,480/128,691) of users visited the Track Progress content area. Within this content area, 13.67% (17,589/128,691) of users completed at least one PCL-5 assessment, and 3.88% (4989/128,691) of users completed 2 or more assessments. In addition, 9.05% (11,647/128,691) of users set a reminder to complete future PCL-5 assessments.

Third, 28.49% (36,666/128,691) of users visited the Learn content area. Within this content area, 16.29% (20,964/128,691) of users viewed at least one learn topic under any category. Under the About PTSD category, 12.55% (16,152/128,691) of users viewed at least one learning topic, with the most frequent topic being PTSD Facts (8904/128,691, 6.92%). Under Getting Professional Help, 3.47% (4463/128,691) of users viewed at least one learn topic, with the most frequent topic being tools for PTSD (2141/128,691, 1.66%). Under PTSD and the Family, 5.12% (6587/128,691) of users viewed at least one learn topic, with the most frequent topic being Fighting Fair (3366/128,691, 2.62%).

Fourth, 21.52% (27,701/128,691) of users visited the Get Support content area. Within this content area, 9.7% (12,491/128,691) of users viewed Crisis Resources, 5.76% (7409/128,691) viewed Find Professional Care, and 6.68% (8600/128,691) viewed Grow Your Support. From 1 of these 3 categories, 3.52% (4535/128,691) of users accessed a website or phone resource. Also within this content area, 4.76% (6129/128,691) of users added personal support contact.

Fifth, regarding the safety plan for suicide prevention, 2.47% (3184/128,691) of users opened the plan, and 0.25% (320/128,691) of users also completed the plan. Of note, the 1100 VA staff who participated in the NCPTSD mHealth app training likely comprised about one-third of the users who opened the plan. It is not clear how many staff completed the plan, given that it was not required as part of the training.

Lastly, when restricting to the subset of high-engagement users identified above (2601/128,691, 2.02% of new users), a much higher proportion of these users visited key app features compared with the proportion among all users. For example, 87.43% (2274/2601) of these high-engagement users accessed at least one coping tool, 55.36% (1440/2601) completed at least one PCL-5 assessment, 52.67% (1370/2601) viewed at least one learn topic, 18.22% (474/2601) accessed a Get Support website or phone resource, and 2.04% (53/2601) completed a safety plan for suicide prevention. These percentages are between 2 and 10 times greater than the percentages for all users.

#### Impact

Among the users who had pretool SUDs ratings (n=56,971), first SUDs ratings reflected relatively high momentary distress (mean 6.03, SD 2.52). Among the users who had both pretool and posttool SUDs ratings (n=17,841), SUDs ratings decreased after using a tool, with a mean difference of −1.38 (SD 1.70; 95% CI −1.41 to −1.36), which was significantly different from 0 (t_17,840_=−108.23; *P*<.001). The mean SUDs decrease was comparable among the subset of high-engagement users who had both pretool and posttool SUDs ratings (n=1447; mean −1.48, SD 1.42). Excluding these high-engagement users from analysis did not impact the mean for the remaining users (mean −1.37, SD 1.73), suggesting that the potential benefit of momentary distress relief was not driven by the high-engagement users.

Among the users who had at least one PCL-5 score (n=17,589), initial scores reflected high levels of PTSD symptoms (mean 49.80, SD 16.36), with 87.13% (15,326/17,589) of these users with a score of ≥31 points (ie, the threshold for likely PTSD diagnosis). Among users with at least two PCL-5 scores (n=4989), scores decreased from the first to last PCL-5, with a mean difference of −4.35 (SD 15.29; 95% CI −4.77 to −3.92) that was significantly different from zero, (t_4988_ =−20.07; *P*<.001). Specifically, 31.77% (1585/4989) of users with at least two PCL-5 scores had clinically significant decreases (ie, ≥10 points), and 44.34% (2212/4989) of users with at least two PCL-5 scores had reliable decreases (ie, ≥5 points) in their scores.

Compared with the mean for all users, the mean PCL-5 decrease was somewhat larger for the subset of high-engagement users who had at least two PCL-5 scores (n=1051; mean −5.95, SD 16.44). Excluding these high-engagement users from analysis slightly attenuated the mean for the remaining users (mean −3.92, SD 14.94). These findings suggest that users as a whole are still experiencing a potential benefit in PTSD symptom reduction and that this benefit may be more pronounced for the subset of high-engagement users.

### Comparison of iOS and Android Users

#### Reach

Among the 207,001 downloads of the PTSD Coach app during the observation period, 73.65% (152,461/207,001) were iOS downloads and 26.35% (54,540/207,001) were Android downloads. Of note, among the 152,461 iOS downloads, 95,000 (62.31%) originated from the VA OCC initiative to provide iOS tablets to veterans engaging in telehealth services. Among the 148,354 total app users, 96,143 (64.81%) were iOS users, and 52,211 (35.19%) were Android users. There was an average of 13,628 active monthly iOS users and 7404 active monthly Android users. Figure 3 displays active monthly users from both platforms for each month during the observation period. In contrast to the number of Android users staying relatively consistent throughout the months, the number of iOS users peaked in the first month before it fell and remained steady, which was likely related to VA OCC striving to meet telehealth demands by distributing iOS tablets during the early months of the COVID-19 pandemic. Lastly, among the 128,691 new users, 80,006 (62.17%) were iOS users, and 48,685 (37.83%) were Android users.

#### Use

In Table 2, the metrics for overall use, use over time, and use of specific features are displayed separately for iOS and Android new users. Across all metrics, iOS users showed lower levels of use compared with Android users. These differences were statistically significant (*P*<.001) and were associated with generally small effect sizes, with the exception of medium effect sizes for number of events per visit, visit duration, and total duration.

**Table 2:** Use of PTSD Coach, version 3.1, among new users between April 20, 2020, and April 19, 2021, separated by platform.

Use category				iOS users (n=80,006)		Android users (n=48,685)		Difference test^a^				Effect size		
								*t* test *(df)*		Chi-square (*df*)		Cohen *d*		Cramer *V*
**Overall use**														
	Number of visits, mean (SD)		3.18 (7.17)		3.39 (7.78)		−3.66 (128,689)		N/A^b^		0.02		N/A	
	Number events per visit, mean (SD)		15.39 (23.57)		22.00 (28.40)		−102.16 (128,689)		N/A		0.61		N/A	
	Visit duration (in minutes), mean (SD)		4.12 (6.67)		5.40 (7.78)		−101.95 (128,689)		N/A		0.64		N/A	
	Total duration (in minutes), mean (SD)		16.03 (53.95)		20.06 (65.50)		−99.52 (128,689)		N/A		0.62		N/A	
	Number of unique days, mean (SD)		2.62 (4.67)		2.83 (5.16)		−5.80 (128,689)		N/A		0.03		N/A	
**Use over time**														
	Days between first and last use, mean (SD)		30.21 (64.33)		33.72 (66.04)		−5.44 (128,689)		N/A		0.03		N/A	
**Use of specific features**														
	Home, n (%)		47,718 (59.64)		37,045 (76.09)		N/A		3643.14 (1)		N/A		0.17	
	Number of content areas, mean (SD)		1.24 (1.40)		1.49 (1.40)		−30.52 (128,689)		N/A		0.18		N/A	
	**Manage Symptoms content area, n (%)**		41,730 (52.16)		29,919 (61.45)		N/A		1060.20 (1)		N/A		0.09	
		Selected symptom	34,396 (42.99)		21,951 (45.09)		N/A		54.02 (1)		N/A		0.02	
		Rated pretool SUDs^c^	33,087 (41.36)		23,884 (49.06)		N/A		727.86 (1)		N/A		0.08	
		Rated pretool and posttool SUDs^c^	7828 (9.78)		10,013 (20.57)		N/A		2946.87 (1)		N/A		0.15	
		Accessed coping tool	31,056 (38.82)		22,258 (45.72)		N/A		594.30 (1)		N/A		0.07	
	**Track Progress content area, n (%)**		20,275 (25.34)		15,205 (31.23)		N/A		525.90 (1)		N/A		0.06	
		Completed 1 PCL-5^d^	10,137 (12.67)		7452 (15.31)		N/A		178.27 (1)		N/A		0.04	
		Completed ≥2 PCL-5s	2785 (3.48)		2402 (4.93)		N/A		232.07 (1)		N/A		0.04	
		Set assessment reminder	6507 (8.13)		5140 (10.56)		N/A		216.20 (1)		N/A		0.04	
	**Learn content area, n (%)**		20,766 (25.96)		15,900 (32.66)		N/A		667.72 (1)		N/A		0.07	
		Accessed learn topic	11,863 (14.83)		9101 (18.69)		N/A		331.82 (1)		N/A		0.05	
	**Get Support content area, n (%)**		16,407 (20.51)		11,294 (23.2)		N/A		129.80 (1)		N/A		0.03	
		Accessed web or phone resource	2346 (2.93)		2189 (4.5)		N/A		217.76 (1)		N/A		0.04	
		Added support contact	2707 (3.38)		3422 (7.03)		N/A		886.82 (1)		N/A		0.08	
	Completed safety plan for suicide prevention, n (%)		162 (0.2)		158 (0.32)		N/A		18.18 (1)		N/A		0.01	

^a^All differences were statistically significant at *P*<.001.

^b^N/A: not applicable.

^c^SUDs: subjective units of distress.

^d^PCL-5: Posttraumatic Stress Disorder Checklist for DSM-5.

#### Impact

Among the iOS (n=33,087) and Android (n=23,884) users who had pretool SUDs ratings, ratings from iOS users (mean 5.85, SD 2.56) reflected lower momentary distress (*P*<.001; Cohen *d*=0.16) than those from Android users (mean 6.26, SD 2.46). Among the iOS (n=7828) and Android (n=10,013) users who had both pretool and posttool SUDs ratings, iOS users (mean −1.70, SD 1.80) had larger reductions (*P*<.001; Cohen *d*=0.33) than Android users (mean −1.14, SD 1.59).

Among the iOS (n=10,137) and Android (n=7452) users who had at least one PCL-5 score, initial scores reflected lower levels of PTSD symptoms (*P*<.001; Cohen *d*=0.06) for iOS users (mean 49.39, SD 16.32) than for Android users (mean 50.36, SD 16.40). Among iOS (n=2590) and Android (n=2399) users with at least two PCL-5 scores, there was a similar decrease in scores (*P*=.26; Cohen *d*=0.03) from the first to last PCL-5 (iOS: mean −4.58, SD 15.45; Android: mean −4.10, SD 15.12).

## Discussion

### Overview

Developed by VA NCPTSD and Department of Defense Center for Telehealth and Technology, PTSD Coach is an evidence-based, secure app that is available for free to the general public for the self-management of PTSD symptoms. In a previous study, version 1 of PTSD Coach was found to have been positively received and had a wide reach among members of the public, who used the app to varying extents and found it helpful in reducing momentary distress [17]. Because PTSD Coach has been updated in the last several years with new features and analysis capabilities, in this study, we examined the reach, use, and potential impact of the current version of PTSD Coach, version 3.1, through utilizing public use data between April 2020 and April 2021. In addition, as part of evaluating use, we were able to extend prior work by characterizing the frequency of use of specific app features in PTSD Coach, thereby establishing a baseline on *how* the app is being used as part of examining engagement [25].

### Principal Findings and Comparison With Previous Work

First, we found that the PTSD Coach app continued to achieve broad dissemination to the general public, with approximately 210,000 downloads, 150,000 total users, 130,000 new users, and 20,000 active users per month during the 1-year data observation period. Among the 210,000 downloads, 95,000 downloads originated from VA OCC’s initiative to distribute iOS tablets that were preloaded with a range of health care apps to veterans. Even after accounting for these institutional downloads, the reach of PTSD Coach appears to be considerably expanded for version 3.1 compared with version 1 (which had approximately 150,000 downloads and 10,000 active users per month during a 3-year observation period [17]).

Second, PTSD Coach, version 3.1, was used, on average, 3 times across 3 separate days for a total duration of 18 minutes of use. Outside of the mean, other values (eg, median, maximum, and IQR) for our use metrics revealed that there was a subset of users with much higher levels of engagement than the average user; we used an example cutoff (ie, ≥2 SDs above the mean for the number of visits) to illustrate the use patterns for this subgroup. In terms of use over time, we observed sharp attrition rates; however, there were also some users who were still using the app 12 months later, indicating potential long-term use. Overall, these metrics demonstrate a similar pattern of use that was previously found with version 1 [17]. There is preliminary evidence, however, that version 3.1 was being used for a longer total duration than version 1 (18 vs 5 minutes, respectively), but we interpret this cautiously, given the different approaches used to define the end of a visit.

For the use of specific features, which we were able to examine for the first time with version 3.1, we found that most users (79,099/128,691, 61.46%) arrived at the home screen and proceeded to a main content area. Among all the content areas, Manage Symptoms was accessed most frequently. Within this content area, over 40% (53,314/128,691, 41.43% to 56,971/128,691, 44.27%; depending on the feature) of users selected a current symptom they wished to address, rated their SUDs, and accessed a coping tool. Users indicated that they most frequently wanted help with PTSD re-experiencing symptoms (ie, Reminded of Trauma), and they frequently accessed tools involving a cognitive restructuring component (ie, Change Your Perspective and Inspiring Quotes); 1 caveat was that these tools were also the most frequently recommended across the different symptoms. In contrast to these Manage Symptoms features, the use of specific features in other content areas and parts of the app was lower (eg, the next highest was 20,964/128,691, 16.29% of users accessing a Learn topic) and was lowest for the safety plan for suicide prevention (with only 320/128,691, 0.25% of users completing the plan). For the safety plan, the actual frequency of use was likely lower, as our numbers were influenced by the NCPTSD training for VA staff on the use of VA mHealth apps. To better increase access to this feature, the NCPTSD is working on building a stand-alone safety plan app.

Third, users of PTSD Coach who provided SUDs ratings or completed self-assessments generally endorsed high levels of momentary distress and high levels of PTSD symptoms with most above clinical threshold. Thus, the reach of the app includes members of the general population who are experiencing difficulties in coping with trauma. In terms of potential impact, the average decrease in momentary distress after coping tool use (approximately 1 point on a scale of 0-10) and the average decrease in PTSD symptoms (approximately 4 points on the PCL-5) were both modest. It is important to note that these averages were calculated from a small proportion of users (eg, 4989/128,691, 3.88%, with at least two PCL-5 assessments). However, even a minority of users in this study still represents a large number of people who experienced potential benefits (eg, approximately one-third, 1585/4989, 31.77% of users with at least two PCL-5 assessments had scores reflecting clinically significant improvement in PTSD symptoms). Comparing across the 2 app versions, users appeared to be similarly distressed. However, when compared with version 1, version 3.1 appears to be associated with slightly attenuated effects for SUDs change after tool use (ie, a 1-point vs 2-point average decrease); it is possible that, as the number of tools increased within the app, or as the number of mHealth apps increased, a user might have experienced less satisfaction even for the same tool (a general phenomenon known as the paradox of choice [37]). Changes in PCL scores were not available in version 1 for comparison with version 3.1. Although the impact on momentary distress may be small, there was a positive trajectory in the reduction of PTSD symptoms over time with version 3.1.

Notably, the positive trajectory in PTSD symptom reduction appears to reflect a potential overall benefit from the app. Average PTSD symptom reduction (approximately 4 points on the PCL-5) for all users with at least two PCL-5 assessments (n=4989) was not primarily driven by a subset of high-engagement users with at least two PCL-5 assessments (n=1051). This subgroup, however, had a slightly greater PTSD symptom reduction (approximately 6 points). Considering the use patterns described above (eg, a mean of 3 visits for 18 minutes of use), we think that most users may be experiencing benefits from the app by practicing coping tools or learning information about PTSD during times of distress. It is our hope that, after discontinuing use of the app, these users can continue to use the coping tools that they learned or are more empowered to make decisions about how to manage PTSD. In contrast to average users, the subset of high-engagement users visited key features of the app more frequently and over an extended period. These users may be incorporating app features as part of a self-care routine; for example, regularly tracking PTSD symptoms by completing self-assessments.

Lastly, we compared the reach, use, and potential impact of PTSD Coach, version 3.1, for iOS and Android users. The app continues to reach more iOS users than Android users, which makes sense given that iOS users make up most smartphone users in the United States [38]. However, iOS users used the app to a lesser extent. iOS users also showed lower levels of momentary distress and PTSD symptoms. There were mixed findings on whether the 2 groups benefited similarly from the app, with larger decreases in pre- to posttool SUDs for iOS users than for Android users but similar decreases in PTSD symptoms. Of note, most of these statistically significant differences (which were not surprising given our large sample size) were associated with small effect sizes, suggesting that any difference in experience for an individual user across the 2 platforms was relatively small. The greater reach, lower use, and lower distress among iOS users could have been shaped by VA OCC’s broad distribution of iOS tablets to veterans (even though veterans themselves may be more likely to own Android smartphones [39]). Some veterans may have opened the app on the tablet but were not motivated to continue to use it because they did not have a particular need for it, in contrast to other veterans or users who searched, found, and installed the app on their own. In fact, approximately only 40% of the veterans receiving a tablet during the observation period had a diagnosis of PTSD (Cindie Slightam, MPH, email communication, October 13, 2021). Taking into account VA OCC’s potential influence on version 3.1 iOS metrics, we may effectively be seeing that differences between the 2 platforms have leveled out over time (because with version 1, there was an opposite pattern with lower rates of use for Android users than for iOS users [17]).

### Limitations and Future Directions

Although our study had several strengths (eg, a large sample size, a naturalistic approach, and the examination of specific app features), it also had the following limitations. Our PTSD Coach data were collected exclusively during the COVID-19 pandemic, which could have limited the generalizability of our findings. We note that version 3.1 and version 1 users endorsed similar levels of PTSD symptoms and that use of version 3.1 did not seem to fluctuate alongside peaks of COVID-19 cases in the country. This increased our confidence that use of PTSD Coach during this time was still linked to self-management of PTSD symptoms, rather than self-management of more general distress.

We were able to shed light on how different features of the app were being used, but these frequency findings could have been influenced by order effects. For example, among the more frequently accessed parts of the app, the Manage Symptoms content area is located in the upper left quadrant of the home screen, and Reminded of Trauma is at the top of the list of symptoms. To draw stronger conclusions about which app features users are attracted to, future research could use A/B testing designs (eg, switching the order of app features and examining the resulting impact).

Owing to the anonymous nature of the data, we did not have information about our users, beyond their completed ratings and self-assessments. We note that, compared with the percentage of users who endorsed clinically significant PTSD symptoms, a lower percentage of users accessed information about getting professional care within the app. It may be helpful to find ways to highlight these resources in the app. However, it is possible that many of these users are already under the care of a mental health professional. In addition to future research investigating the treatment status of PTSD Coach users, it would be valuable to know the characteristics of users (eg, demographics, veteran status, and trauma history) who experienced clinically significant symptom improvements. For example, we would hope to see that users in this group include both veterans with histories of combat trauma or military sexual trauma as well as nonveterans experiencing other types of trauma (eg, motor vehicle accidents). Gaining traction in the literature, the precision medicine endeavor to answer, “What works well for whom?” [40] should include testing self-management mHealth apps as an intervention format that may be a particularly good fit for certain individuals. Matching people appropriately to using a self-management app could potentially reduce the strain on the mental health system and allow providers to maximize their time (eg, in this case, possibly allowing for the reallocation of 476 direct clinical care hours, if multiplying the n=1585 with clinically significant PTSD symptom improvement by 18 minutes of app use).

Finally, although the raw use data contained timestamps for individual events, the summary of metrics extracted for each user was not in a longitudinal format. Thus, we could not examine the order in which users engaged with different features. For example, we extracted first and last PCL-5 assessment scores, but we did not know when these self-assessments occurred relative to other events in the app. Having a longitudinal data set in which the use of key features is logged in chronological order would enable researchers to better investigate questions of how to optimize engagement with the app. Given that there was a subset of high-engagement users in PTSD Coach, which was consistent with naturalistic studies of other VA self-management apps [34,41], future research could investigate factors that are associated with increased engagement [42] to try to underscore these factors in the app. One such factor could be the completion of a self-assessment upon first using an app, as this was recently linked to using an app on more days as well as using more coping tools, within the COVID Coach app [41].

### Conclusions

In summary, we found evidence that PTSD Coach, version 3.1, is serving its intended purpose as a public health resource. The app reached a large number of people, including those who were experiencing significant levels of PTSD symptoms (ie, the target population), and likely expanded access to evidence-based interventions and resources. Most users visited the app only a few times but engaged with key app content. Some app features (eg, coping tools) were accessed more frequently than others (eg, self-assessments), giving researchers a sense of what was appealing to users and what could potentially be improved within the app. Although benefits in momentary distress and PTSD symptoms were generally small on a per-individual basis, the app made these benefits available to the population on a large scale, which could have resulted in a cumulative, positive impact on public health (ie, with impact defined as the product of reach and efficacy [43]). Future research should aim to more flexibly examine the utility of different app features (eg, through A/B testing), as well as to investigate questions on understanding effectiveness (eg, to better match the intervention format to the person) and optimizing engagement (eg, to enhance the likelihood of a meaningful impact) with the app. Pursuing research through these avenues will help to ensure that mHealth apps can reach their full potential to alleviate symptoms and to enhance well-being and functioning for individuals with PTSD.

## Appendix

Multimedia Appendix 1 List of coping tools and learn topics in the PTSD Coach Version 3.1 mobile app.

## Abbreviations

EULA: End User License Agreement
mHealth: mobile health
NCPTSD: National Center for PTSD
OCC: Office of Connected Care
PCL-5: Posttraumatic Stress Disorder Checklist for DSM-5
PTSD: posttraumatic stress disorder
SUDs: subjective units of distress
VA: Department of Veterans Affairs

## References

[1] Mobile fact sheet. Pew Research Center.

[2] Price M, Yuen EK, Goetter EM, Herbert JD, Forman EM, Acierno R, Ruggiero KJ (2014). mHealth: a mechanism to deliver more accessible, more effective mental health care. Clin Psychol Psychother.

[3] Demyttenaere K, Bruffaerts R, Posada-Villa J, Gasquet I, Kovess V, Lepine JP, Angermeyer MC, Bernert S, de Girolamo G, Morosini P, Polidori G, Kikkawa T, Kawakami N, Ono Y, Takeshima T, Uda H, Karam EG, Fayyad JA, Karam AN, Mneimneh ZN, Medina-Mora ME, Borges G, Lara C, de Graaf R, Ormel J, Gureje O, Shen Y, Huang Y, Zhang M, Alonso J, Haro JM, Vilagut G, Bromet EJ, Gluzman S, Webb C, Kessler RC, Merikangas KR, Anthony JC, Von Korff MR, Wang PS, Brugha TS, Aguilar-Gaxiola S, Lee S, Heeringa S, Pennell B, Zaslavsky AM, Ustun TB, Chatterji S, WHO World Mental Health Survey Consortium (2004). Prevalence, severity, and unmet need for treatment of mental disorders in the World Health Organization World Mental Health Surveys. JAMA.

[4] Eylem O, de Wit L, van Straten A, Steubl L, Melissourgaki Z, Danışman GT, de Vries R, Kerkhof AJ, Bhui K, Cuijpers P (2020). Stigma for common mental disorders in racial minorities and majorities a systematic review and meta-analysis. BMC Public Health.

[5] (2001). Mental Health Culture, Race, and Ethnicity : A Supplement to Mental Health : A Report of the Surgeon General · Volume 2.

[6] Anthes E (2016). Pocket psychiatry: mobile mental-health apps have exploded onto the market, but few have been thoroughly tested. Nature.

[7] Torous J, Firth J, Huckvale K, Larsen ME, Cosco TD, Carney R, Chan S, Pratap A, Yellowlees P, Wykes T, Keshavan M, Christensen H (2018). The emerging imperative for a consensus approach toward the rating and clinical recommendation of mental health apps. J Nerv Ment Dis.

[8] Wang K, Varma DS, Prosperi M (2018). A systematic review of the effectiveness of mobile apps for monitoring and management of mental health symptoms or disorders. J Psychiatr Res.

[9] Mohr DC, Cheung K, Schueller SM, Hendricks Brown C, Duan N (2013). Continuous evaluation of evolving behavioral intervention technologies. Am J Prev Med.

[10] Holowka D, Marx B (2012). Assessing PTSD-related functional impairment and quality of life. The Oxford Handbook of Traumatic Stress Disorders.

[11] Schnurr PP, Lunney CA, Bovin MJ, Marx BP (2009). Posttraumatic stress disorder and quality of life: extension of findings to veterans of the wars in Iraq and Afghanistan. Clin Psychol Rev.

[12] Kessler RC, Berglund P, Demler O, Jin R, Merikangas KR, Walters EE (2005). Lifetime prevalence and age-of-onset distributions of DSM-IV disorders in the National Comorbidity Survey Replication. Arch Gen Psychiatry.

[13] Wisco BE, Marx BP, Wolf EJ, Miller MW, Southwick SM, Pietrzak RH (2014). Posttraumatic stress disorder in the US veteran population. J Clin Psychiatry.

[14] Owen JE, Kuhn E, Jaworski BK, McGee-Vincent P, Juhasz K, Hoffman JE, Rosen C (2018). VA mobile apps for PTSD and related problems: public health resources for veterans and those who care for them. Mhealth.

[15] Beck A, Rush A, Shaw B, Emery G (1979). Cognitive Therapy of Depression.

[16] Kuhn E, van der Meer C, Owen JE, Hoffman JE, Cash R, Carrese P, Olff M, Bakker A, Schellong J, Lorenz P, Schopp M, Rau H, Weidner K, Arnberg FK, Cernvall M, Iversen T (2018). PTSD Coach around the world. Mhealth.

[17] Owen JE, Jaworski BK, Kuhn E, Makin-Byrd KN, Ramsey KM, Hoffman JE (2015). mHealth in the wild: using novel data to examine the reach, use, and impact of PTSD coach. JMIR Ment Health.

[18] Baumel A, Muench F, Edan S, Kane JM (2019). Objective user engagement with mental health apps: systematic search and panel-based usage analysis. J Med Internet Res.

[19] Kuhn E, Greene C, Hoffman J, Nguyen T, Wald L, Schmidt J, Ramsey KM, Ruzek J (2014). Preliminary evaluation of PTSD Coach, a smartphone app for post-traumatic stress symptoms. Mil Med.

[20] Miner A, Kuhn E, Hoffman JE, Owen JE, Ruzek JI, Taylor CB (2016). Feasibility, acceptability, and potential efficacy of the PTSD Coach app: a pilot randomized controlled trial with community trauma survivors. Psychol Trauma.

[21] Keen SM, Roberts N (2017). Preliminary evidence for the use and efficacy of mobile health applications in managing posttraumatic stress disorder symptoms. Health Systems.

[22] Kuhn E, Kanuri N, Hoffman JE, Garvert DW, Ruzek JI, Taylor CB (2017). A randomized controlled trial of a smartphone app for posttraumatic stress disorder symptoms. J Consult Clin Psychol.

[23] Possemato K, Kuhn E, Johnson E, Hoffman JE, Owen JE, Kanuri N, De Stefano L, Brooks E (2016). Using PTSD Coach in primary care with and without clinician support: a pilot randomized controlled trial. Gen Hosp Psychiatry.

[24] American Psychiatric Association (2013). Diagnostic and Statistical Manual of Mental Disorders: Dsm-5™.

[25] Zhang R, Nicholas J, Knapp AA, Graham AK, Gray E, Kwasny MJ, Reddy M, Mohr DC (2019). Clinically meaningful use of mental health apps and its effects on depression: mixed methods study. J Med Internet Res.

[26] Vindegaard N, Benros ME (2020). COVID-19 pandemic and mental health consequences: systematic review of the current evidence. Brain Behav Immun.

[27] Zulman D, Wong E, Slightam C, Gregory A, Jacobs JC, Kimerling R, Blonigen DM, Peters J, Heyworth L (2019). Making connections: nationwide implementation of video telehealth tablets to address access barriers in veterans. JAMIA Open.

[28] Miliard M VA working with Apple to broaden telehealth access for veterans. Healthcare IT News.

[29] PTSD: National Center for PTSD. U.S. Department of Veterans Affairs.

[30] Weathers F, Litz BT, Keane TM, Palmieri PA PA, Marx  BP,, Schnurr PP The PTSD Checklist for DSM-5 (PCL-5). National Center for PTSD.

[31] Blevins CA, Weathers FW, Davis MT, Witte TK, Domino JL (2015). The posttraumatic stress disorder checklist for DSM-5 (PCL-5): development and initial psychometric evaluation. J Trauma Stress.

[32] Bovin MJ, Marx BP, Weathers FW, Gallagher MW, Rodriguez P, Schnurr PP, Keane TM (2016). Psychometric properties of the PTSD Checklist for Diagnostic and Statistical Manual of Mental Disorders-Fifth Edition (PCL-5) in veterans. Psychol Assess.

[33] Stanley B, Brown GK (2012). Safety planning intervention: a brief intervention to mitigate suicide risk. Cognit Behav Practice.

[34] Kozlov E, Bantum E, Pagano I, Walser R, Ramsey K, Taylor K, Jaworski B, Owen J (2020). The reach, use, and impact of a free mHealth mindfulness app in the general population: mobile data analysis. JMIR Ment Health.

[35] McKinney W (2010). Data structures for statistical computing in Python. Proceedings of the 9th Python in Science Conference.

[36] Project description for pyodbc. pyodbc.

[37] Morita P (2020). Design of mobile health technology. Design for Health Applications of Human Factors.

[38] Wallen J Why is Android more popular globally, while iOS rules the US?. Tech Republic.

[39] Edwards-Stewart A, Smolenski DJ, Reger GM, Bush N, Workman DE (2016). An analysis of personal technology use by service members and military behavioral health providers. Military Med.

[40] Bickman L, Lyon AR, Wolpert M (2016). Achieving precision mental health through effective assessment, monitoring, and feedback processes : introduction to the special issue. Adm Policy Ment Health.

[41] Jaworski BK, Taylor K, Ramsey KM, Heinz A, Steinmetz S, Pagano I, Moraja G, Owen JE (2021). Exploring usage of COVID coach, a public mental health app designed for the COVID-19 pandemic: evaluation of analytics data. J Med Internet Res.

[42] Borghouts J, Eikey E, Mark G, De Leon C, Schueller SM, Schneider M, Stadnick N, Zheng K, Mukamel D, Sorkin DH (2021). Barriers to and facilitators of user engagement with digital mental health interventions: systematic review. J Med Internet Res.

[43] Abrams D, Orleans C, Niaura R, Goldstein M, Prochaska J, Velicer W (1996). Integrating individual and public health perspectives for treatment of tobacco dependence under managed health care: a combined stepped-care and matching model. Ann Behav Med.

