# Supplementary material for: PTSD Coach Version 3.1: A Closer Look at the Reach, Use, and Potential Impact of This Updated Mobile Health App in the General Public
Source: JMIR Ment Health. 2022 Mar 29;9(3):e34744. doi: 10.2196/34744 (PMC9006138; doi:10.2196/34744)
Supplement: Multimedia Appendix 1 [file mental_v9i3e34744_app1.docx]

Multimedia Appendix

PTSD Coach Version 3.1 Mobile App

Coping tools from the Manage Symptoms content area

1. Ambient sounds
2. Body scan
3. Change your perspective
4. Connect with others
5. Deep breathing
6. Grounding
7. Inspiring quotes
8. Leisure activities
9. Mindfulness
10. Muscle relaxation
11. My feelings
12. Observe thoughts
13. Positive imagery
14. Relationship tools
15. RID (Relax, Identify, Decide): Coping with triggers
16. Schedule worry time
17. Seeing my strengths
18. Sleep tools
19. Soothe the senses
20. Soothing audio
21. Soothing images
22. Thought shifting
23. Time out

About PTSD topics from the Learn content area

1. What is PTSD?
2. PTSD facts
3. How does PTSD develop?
4. How common is PTSD?
5. Who develops PTSD?
6. How long does PTSD last?
7. Problems related to PTSD
8. Understanding PTSD treatment
9. Do I have PTSD?
10. I have PTSD…
11. I’m embarrassed to have PTSD
12. Social isolation
13. Sleep problems: Nightmares
14. Sleep problems: Insomnia
15. What do I do if I get triggered?
16. What is dissociation?
17. I am avoiding things
18. I don’t trust people
19. I can’t control my temper
20. I’m always on edge
21. I feel sad all the time

Getting Professional Help topics from the Learn content area

1. I’m in a crisis
2. Treatment locator
3. Finding treatment for alcohol or drugs
4. What is counseling (therapy)?
5. Tools for PTSD
6. How do I find a counselor/therapist?
7. Do I need professional help?
8. Why do people seek counseling?
9. How can a therapist help me?
10. Is counseling confidential?
11. Will therapy really work?
12. PTSD treatments that work
13. How much does counseling cost?
14. I want counseling, but I work all day
15. Transportation to appointments
16. I’m embarrassed to go for counseling
17. Who can help me?
18. What does a primary care physician do?
19. What does a social worker do?
20. What does a psychologist do?
21. What does a psychiatrist do?
22. What does a pastoral counselor or chaplain do?

PTSD and the Family topics from the Learn content area

1. What is couples counseling?
2. What is family therapy?
3. Reconnecting with your partner
4. Fighting fair
5. Talking to kids about PTSD
6. Impact of PTSD on children
7. Children’s responses to PTSD symptoms
8. Can children get PTSD from their parents?
9. Helping children cope
10. Parenting tips
11. Are my kids ok? When to seek outside help
12. Should my child have individual counseling?
